# Occupational physical activity, all-cause mortality and incidence of cardiovascular diseases: results from three Italian cohorts

**DOI:** 10.1007/s00420-023-02028-w

**Published:** 2023-12-15

**Authors:** Dario Fontana, Raffaele Ceron, Angelo d’Errico

**Affiliations:** 1 Epidemiology Unit, ASL TO3, Piedmont Region, Grugliasco, Turin Italy; 2 Occupational Health and Safety Unit, ASL CN1, Piedmont Region, Saluzzo, Cuneo Italy

**Keywords:** Occupational physical activity, Mortality, Cardiovascular diseases, Longitudinal studies, Job-exposure matrix, Epidemiology

## Abstract

**Purpose:**

To examine the association of exposure to Occupational Physical Activity (OPA) with all-cause mortality and incidence of cardiovascular diseases (CVD).

**Methods:**

The study population was composed of three Italian cohorts: a national cohort of employees participating in the National Health Survey 2005, followed-up until 2014 (ILS 2005), and two urban cohorts of employees resident in Turin at 2001 and 2011 censuses (TLS 2001 and TLS 2011, respectively), both followed-up until 2018. Follow-up was conducted through individual record-linkage with death registries and hospital admissions archives. Exposure to OPA was assigned through an Italian job-exposure matrix (JEM). Relative Risks of both CVD incidence and overall mortality associated with OPA quartiles (IRR) were estimated using Poisson regression models adjusted for socio-demographics and health, and in the national cohort, also for leisure time physical activity, BMI, smoking, diabetes, and hypertension.

**Results:**

Compared to the lowest quartile, the highest OPA quartile was associated in both genders with significantly increased mortality in TLS 2001 (IRR = 1.11 among men, IRR = 1.20 among women) and in TLS 2011 (IRR = 1.27 among men and IRR = 1.73 among women), whereas in the ILS 2005 cohort no association was found. Among women, high OPA was also associated with CVD risk in TLS 2001 and 2011 (IRR = 1.39 and IRR = 1.16 for the highest quartile, respectively), while in the ILS cohort in both genders only the third quartile showed a significantly higher risk.

**Conclusion:**

Our results indicate that OPA does not have a beneficial effect on CVD and mortality, but rather suggest that it may produce deleterious health effects.

**Supplementary Information:**

The online version contains supplementary material available at 10.1007/s00420-023-02028-w.

## Introduction

Although for leisure time physical activity (LTPA) beneficial effects on health have been consistently shown, including a decrease in mortality and in cardiovascular diseases (CVD) incidence (Cheng et al. 2018), for occupational physical activity (OPA) the association with these health outcomes appears mixed in the literature. Earlier studies from the ‘50 s and the ‘70 s found higher longevity among workers exposed to high OPA (Morris et al. 1953; Kagan 1976; Rose and Cohen 1977), whereas most of more recent studies have rather found no significant association or an increased risk of mortality. A meta-analysis showed an 18% increase in mortality among men employed in jobs with high OPA, compared to those in jobs with low OPA, but no significant association was demonstrated among women (Coenen et al. 2018). Regarding CVD, in a meta-analysis of seven cohort studies the risk of CVD was 25% higher for exposure to high vs. low OPA (Li et al. 2013), whereas a recent systematic review did not find any association between high OPA and CVD mortality in men or women (Cillekens et al. 2023). Furthermore, two recent large longitudinal studies with thorough adjustment for potential confounders found opposite results: while in a Norwegian study men exposed to high OPA showed significantly higher longevity (Dalene et al. 2021), in a Danish study both CVD incidence and all-cause mortality were increased for exposure to high OPA (Holtermann et al. 2021). Other recent studies reported an increased risk of mortality or CVD associated with high OPA in various countries (Martinez-Gomez et al. 2022; Hermansen et al. 2019; Wanner et al. 2019; Quinn et al. 2021), although several other studies did not find any association (Luo et al. 2022; Pearce et al. 2021; Strippoli et al. 2022; Bonekamp et al. 2022).

In spite of the mounting evidence on the possible negative health effects of OPA, the last WHO guidelines on physical activity still equate the health benefits of LTPA with those of OPA, stating that “every step counts” in reaching the physical activity amount recommended, independently if performed in leisure time or at work; in fact, according to the WHO guidelines, there is insufficient evidence to distinguish between the effects on health of physical activity in different domains (Bull et al. 2020).

The discrepancy between the detrimental effect of OPA on health and the beneficial one of LTPA has been named the “physical activity paradox” and interpreted as due to differences in the characteristics of physical activity in the two domains: compared to LTPA, OPA would involve in general lower intensity levels of activity, mainly unable to improve cardiorespiratory fitness, but sustained for much longer time, often with insufficient recovery pauses (Holtermann et al. 2018).

However, it has been commented that the positive association between OPA and mortality could have been determined by confounding (or residual confounding) by socioeconomic position (SEP) or health status, as higher OPA and poorer health are more diffuse in workers in more disadvantaged social classes, and in many studies results have been adjusted only for age, gender and for a socioeconomic- or a health-related covariate (Coenen et al. 2020). Possible confounding by lifestyles or exposure to other work hazards eventually correlated with higher OPA, such as workplace air pollutants and psychosocial factors, has been also suggested (Reinhardt et al. 2013). Another possible source of inconsistency of the results in the literature is that the association of OPA with mortality and CVD may be influenced by the level of LTPA, as reported by some studies, where OPA was associated with a higher risk in presence of low LTPA (Clays et al. 2013; Harari et al. 2015).

Furthermore, given that exposure to OPA was self-reported in almost all studies on mortality or CVD, differential misclassification of exposure to OPA by case status is another possible bias which could have led to a spurious positive association with OPA, not only in cross-sectional and case–control studies but also in longitudinal studies in which health status at baseline had not been accurately adjusted for. On the other hand, as most studies assessed exposure to OPA only once in time, instead of cumulative exposure during working life, there is the possibility that the association with health outcomes has been underestimated, due to non-differential exposure misclassification. Health selection of workers into less exposed job or into retirement could have also contributed to underestimate the association with OPA (Flower et al. 2019).

With the present study, we wanted to contribute to the current debate on the health effects of OPA, evaluating the impact of exposure to high OPA, assessed through a Job-Exposure Matrix (JEM) and not by self-reports as in previous studies, on the risk of both all-cause mortality and CVD incidence in three large cohorts, which are among the largest longitudinal datasets available in Italy. All three had available information on several potential confounders, including socio-demographic characteristics and health status, and in one also behavioural factors. An important feature of the use of JEM for exposure assessment is that it is independent from individuals’ self-reported health status, so that it prevents from differential misclassification of the exposure (Peters 2020). However, non-differential exposure misclassification may be increased, especially for those jobs with higher inter-individual variability in tasks performance and duration, because the JEM assigns the same exposure value to all workers holding the same job title, without accounting for individual differences, which may lead to an underestimation of the exposure‐outcome association. Given that we have conducted a previous study on overall mortality and coronary heart disease incidence on one of these cohorts (Strippoli et al. 2022), in which exposure to OPA was self-reported at baseline, it is of particular interest the comparison of the results of that study with those of the present one, which may help clarifying if differences in methodology of exposure assessment employed may influence the association estimated between OPA and mortality. It appears worth noting that the only two previous studies on mortality which used a JEM for assigning exposure to OPA, both Scandinavian, found a significantly increased risk associated with high OPA (Ervasti et al. 2019; Mikkola et al. 2019). Furthermore, the availability in one of the cohorts on longitudinal information on jobs held across many years allowed estimating a cumulative dose of exposure to OPA of cohort members and examining its impact on the health outcomes considered.

## Material and methods

### Data collection

The study used data from three different cohorts, the first one including participants in the 2005 National Health Interview Survey (NHIS), and the other two consisting of subjects resident in Turin and participating in the censuses 2001 or 2011, respectively.

The NHIS 2005 was conducted on a representative sample of the Italian population, including 50,474 families and 128,040 individuals, with 83% participation. The survey provides detailed information on individual and household socioeconomic status, employment, occupation, lifestyles, and health conditions. Follow-up of the sample for mortality and CVD incidence was conducted by means of record-linkage with the National Registry of Mortality of the National Institute of Statistics (Istat) and with the National Hospital Discharge Database of the Italian Health’s Ministry, respectively. The follow-up of this sample, together with that of the NHIS 2000, also going until 2014, is known as the Italian Longitudinal Study (ILS) (Sebastiani et al. 2019). NHIS 2000 data were not included in the study, because the job classification used in that survey was a previous one (CP1991), for which a crosswalk to translate job codes into those used in the job classification on which the JEM has been constructed (CP2011) is not available.

The second and third datasets are part of the Turin Longitudinal Study (TLS), which is a system of archives containing socio-demographic (data from censuses and civil registries) and health information (hospitalizations, drug prescriptions, mortality, etc.), record-linked at the individual level. 832,106 subjects participated in the 2001 census (TLS 2001 cohort) and 849,686 subjects in the 2011 census (TLS 2011 cohort).

In the national cohort (ILS 2005) and in TLS 2001 cohort, only subjects reporting to work as employees at baseline, and with information on job title, were selected. Regarding the TLS 2011 cohort, as only very crude information was collected on occupation at 2011 census (10 types of occupations in total), the job held was assigned through individual record-linkage to another administrative data source, the regional archive of Compulsory Communications (CC) of start/end job contracts. In this dataset are present all communications sent by employers from 2009 to 2018 to the Regional Employment Office, to report any change occurred in a work contract (hiring, firing, retirement), together with the code of the job held and its date of start and end. Therefore, these data allow the reconstruction of employees’ careers along many years for all subjects who started working, changed job or retired during 2009–2018, with retrospective information going back in many cases until the ‘80 s. Only workers who started a new job after 1995 were included in the study, as the JEM used to assign exposure to OPA was based on data collected in 2013, so it was not considered to represent reliably exposure in jobs held before mid- ‘90 s, when work intensity was generally lower than in later periods in most industries and workplaces, especially in terms of work pace and effort (Green et al. 2001).

In all cohorts, workers had to be between 25 years and the minimum age for pension seniority in the year of enrolment (56 years in 2001, 57 years in 2004, 60 years in 2011): the exclusion of older workers was meant to reduce the possible health selection into retirement of subjects with high exposure to ergonomic factors, which could have caused an underestimation of the true association between OPA and the outcomes.

In the ILS 2005 and the TLS 2001 cohorts, information on the job held at baseline was coded according to the 4-digit Istat 2001 classification of occupations (CP 2001), which includes 519 jobs, while no information was present on previous work history. Job codes of employed subjects in the ILS 2005 cohort were assigned automatically by Istat based on self-reported job title, and revised by one of the authors (R.C.). For TLS 2001, job codes were also assigned automatically by Istat and underwent, a decade ago, a thorough process of revision, funded by Istat, in which one of the authors was also involved (A.D). In contrast, for the TLS 2011 cohort job codes used had been assigned directly by the Regional Employment Office, with no possibility for us to review them, and were based on the 5-digit Istat 2011 classification of occupations (CP 2011), consisting of 796 occupations.

**Fig. 1:**
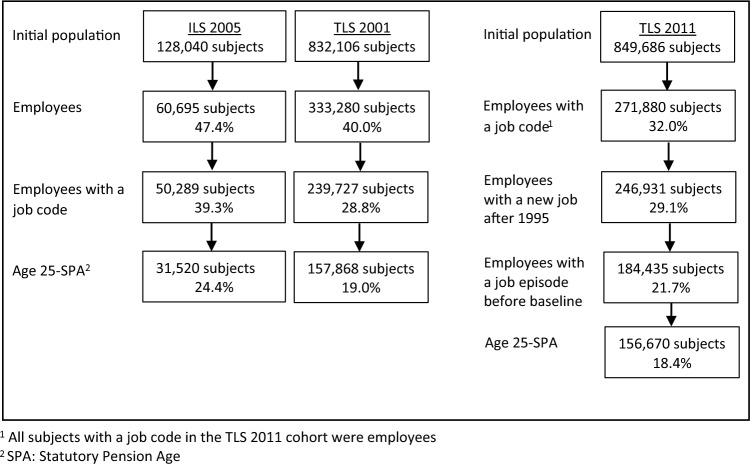
Flowchart of the enrolment of the study population in each cohort

Based on these inclusion criteria, 31,520 subjects were enrolled in the 2005 ILS cohort, 157,868 in the TLS 2001 cohort, and 156,674 in the TLS 2011 cohort. A flowchart showing the steps of the selection of the study population of the three cohorts is presented in Fig. 1.

Subjects in the three cohorts were followed-up for overall mortality and for incidence of CVD, based on hospitalizations and death records. Incidence of CVD was assessed through a first hospital admission (identified by ICD-IX codes 390-459) or mortality for CVD (identified by ICD-X codes I00-I99) during follow-up. In the analyses on CVD, prevalent cases occurred before baseline were excluded. In TLS 2001 and TLS 2011, CVD prevalent cases were excluded based on previous hospital admissions for this disease group from 1995 to start of follow-up (5562 subjects in TLS 2001; 8417 subjects in TLS 2011). In ILS 2005, subjects were identified as CVD prevalent cases and excluded if they reported at baseline to have been diagnosed by a physician with myocardial infarction, angina pectoris, other cardiac diseases, or stroke (*n* = 790).

For the ILS cohort, follow-up started on the date of the baseline interview and ended at the end of 2014, or earlier in case of death or first hospitalization for CVD (for analyses on CVD); for the TLS 2001 and the TLS 2011 cohorts, it started at the beginning of 2002 and 2012, respectively, and ended at the end of 2018, or at the date of death, emigration out of town, or first hospitalization for CVD, if earlier. The TLS 2001 and 2011 cohorts partially overlapped, with 31,448 subjects present in both cohorts.

#### Exposure assessment

Exposure to OPA was assigned to workers in the study through a job-exposure matrix (JEM) constructed from the Italian O*NET database, that contains information on hundreds of physical and mental descriptors, in terms of skills, knowledge, activities, work context, etc., aggregated at the job level (http://www.onetcenter.org). The Italian O*NET database is constructed from workers’ self-reports, based on interviews of approximately 20 workers of any gender for each of the 796 jobs of the Italian classification (CP2011, 5-digit level). For each job, the O*NET database contains scores for each descriptor, rated by importance, frequency, or intensity of a certain workplace characteristic. Answers to these questions are collected on 5-point or 7-point level, depending on the item, and averaged for each of the 796 occupations, with the mean representing the exposure score of a certain job.

From the 100 variables available in O*NET, a JEM was constructed for 21 physical factors, which were further reduced through Principal Component Analysis to 17 factors potentially involving high physical workload. For all 17 factors, good concordance against the same items of a corresponding O*NET JEM created in the United States has been shown (d’Errico et al. 2022a). Of the 17 items, 3 focused on force exertion (static strength; dynamic strength; trunk strength), 6 on activity level and repetitive movements of the upper limb (manual dexterity; fingers dexterity; wrist-finger speed; handling and moving objects; time spent making repetitive motions; time spent using hands to handle, control, or feel objects, tools or controls), 4 on postures (awkward positions; standing; kneeling, crouching, stooping, or crawling; bending and twisting the body), 2 items on activities involving the whole body (performing generalized physical activity; walking and running), 2 items on exposure to vibration (whole-body vibration, driving vehicles or other types of moving machinery). Scores of each item were standardized on a 0–100 scale and averaged, to compute a composite ergonomic exposure index (Ergo-index) (Cronbach alpha = 0.90), which is the measure of exposure to OPA used in this study. Further details on the construction of the JEM and the Ergo-index can be found elsewhere (Fontana et al. 2022; d’Errico et al. 2023).

As job codes in the JEM and in the regional archive of Compulsory Communications (CC) corresponded, being both based on the 2011 classification of occupations (CP 2011), it was possible to assign directly the Ergo-index computed for each 5-digit occupation to subjects in the TLS 2011 cohort. In contrast, job codes in the ILS 2005 and TLS 2001 cohorts were based on a previous job classification (CP 2001), which had to be translated by one of the authors (D.F.) into the CP 2011 one, to be able to assign the Ergo-index to subjects in these cohorts.

For ILS 2005 and TLS 2001, the Ergo-index scores were assigned to the job performed at baseline, as this was the only job known, while for the TLS 2011 cohort the Ergo-index was computed cumulatively from 1995 and over the years of follow-up, summing exposure scores across days of employment for each job held.

### Data analysis

The association of OPA with mortality and CVD incidence was examined both treating the Ergo-index as a continuous measure and dividing it in quartiles (the baseline Ergo-index, for the ILS 2005 and the TLS 2001 cohorts, and the cumulative Ergo-index, for TLS 2011).

Incidence Rate Ratios (IRR) of mortality and CVD incidence associated with exposure quartile were estimated through Poisson regression models stratified by gender. All the analyses were adjusted for age, health, and household typology (single, coupled with children, coupled without children, single parent). In the ILS 2005 study, there was also information on geographical area, economic situation, lifestyles and biological risk factors for CVD, which allowed to perform analyses adjusted also for area of residence (North–West, North–East, Center, South), household economic resources (two categories: excellent or adequate, scarce or absolutely insufficient), smoking (0, 0.1–10, 10.1–20, 20.1–30, > 30 pack-years), BMI (normal or underweight, overweight, obese), leisure time physical activity (LTPA) (no activity, light, regular, intense activity), hypertension (yes/no), and diabetes (yes/no) at baseline. In TLS 2001 and 2011, information on behavioural and biological risk factors for CVD was lacking, but it was possible to further control the analyses for an area deprivation index at the census tract level (Rosano et al. 2021). The health indicator used as a covariate in the analyses on ILS 2005 was the Physical Component Summary, obtained from the SF-12 questionnaire (Ware et al. 1996; Apolone et al. 2001), whereas that used for the analyses on TLS 2001 and TLS 2011 was the Charlson index (Quan et al. 2005), constructed at baseline from cause-specific hospitalizations of the cohorts during the years 1997–2001 and 2007–2011, respectively.

In the analysis of the TLS 2011 cohort, cumulative exposure was treated as a time-varying variable, for which it was estimated the risk of developing the outcome in a certain year of follow-up according to the cumulative exposure occurred until the end of the previous year. In this cohort there was also information about part-time work, which was used to weight cumulative exposure, as well as on having multiple jobs simultaneously, for which exposure was averaged across the different jobs. Periods of unemployment were assigned zero exposure.

Because, especially in ILS 2005 and TLS 2001 data, there was a strong correlation between educational level and exposure to the Ergo-index (ILS 2005: men Spearman rho = 0.55, women rho = 0.51; TLS 2001: men rho = 0.58, women rho = 0.43), there was concern that including both of them in a single regression model could have produced risk estimates affected by multicollinearity, with the possible consequence of an artificial underestimation of the coefficients and of an overestimation of their standard errors (Vatcheva et al. 2016). Therefore, in a first analysis (Model 1) education was not included among covariates, but an adjustment for this variable was performed only in a subsequent analysis (Model 2). Only for ILS 2005, a further analysis included among covariates also the lifestyles and the biological risk factors for CVD described above (Model 3). Trends in risks across Ergo-index quartiles were estimated treating quartiles as continuous variables.

All models were adjusted for health status at baseline, assuming a confounder’s role of health rather than a mediator one on the association between OPA and mortality or CVD, to keep a conservative approach on the possible impact of OPA on the health outcomes. However, a preliminary analysis of the correlation between exposure to OPA and health scores (PCS or Charlson Index) showed that it was quite low (Sperman rho < 0.10 in all cohorts in both genders), so it was expected that the adjustment for health had little influence on the associations investigated.

A Directed Acyclic Graph (DAG) on the hypothesized relationships among the variables included in the analyses on OPA and CVD in ILS 2005 (as this is the cohort for which more covariates were available), is presented in Supplementary Fig. 1.

In the light of the reported effect modification by LTPA of the association between OPA and mortality or CVD risk in the literature, in the ILS 2005 cohort we also tested the interaction between OPA and LTPA dichotomized (no physical activity; light, regular or intense physical activity) in fully adjusted models and performed analyses stratified by LTPA.

Given that the two TLS cohorts were partially overlapping, a sensitivity analysis was also performed on the TLS 2001 cohort after excluding subjects present also in the TLS 2011 cohort.

For all analyses, associations with *p* values below 0.05 (two-tails) were considered statistically significant.

## Results

Characteristics of the three study populations are presented in Table 1. In all cohorts mean age at baseline was the same for men and women (41 years in ILS 2005, 39 years in TLS 2001, and 40 years in TLS 2011). In all cohorts, men were less educated than women, with low-educated women (elementary or low secondary) around 45% in the different cohorts, and men ranging from 50 to 58%. The most diffused household typology was “couple with children”, which included 70% men and 65% women in ILS 2005, although lower proportions among Turin residents, especially in TLS 2011 (49% in men and 43% in women).

**Table 1:** Frequency distribution of the characteristics of the three cohorts, cumulative mortality, and person-days of follow-up, by gender

Variables	ILS 2005		TLS 2001		TLS 2011	
	Men	Women	Men	Women	Men	Women
*Age at baseline (mean—sd)*	41.1 (9.2)	41.0 (9.0)	39.3 (8.7)	39.2 (8.5)	39.8 (9.6)	39.8 (9.4)
*Charlson index (% subjects* > *0)*			3.3	2.6	3.0	2.5
*Deprivation index. (mean—sd)*			0.11^1^ (2.0)	-0.08^1^ (1.9)	1.64^2^ (2.1)	1.48^2^ (2.0)
*Cumulative mortality (%)*	2.2	1.1	4.1	2.3	1.6	0.8
*N. subjects*	15,757	15,763	79,103	78,765	75,618	81,052
*Person-days of follow-up (mean—sd)*	3498.4 (283.3)	3516.8 (210.3)	5185.5 (1842.9)	5375.9 (1728.3)	2289.2 (606.3)	2327.9 (571.3)
*Physical Component Summary (mean—sd)*	53.1 (6.5)	52.2 (7.3)				
*Educational level (%)*						
University	11.5	14.8	17.8	21.9	20.0	23.5
High school	30.6	34.6	32.7	35.3	30.0	32.6
Low secondary/elementary	57.8	50.6	49.5	42.8	50.0	43.9
*Household typology (%)*						
Single	12.6	9.8	17.9	16.0	26.4	24.1
Couple without children	11.0	12.8	16.9	18.0	16.1	16.5
Couple with children	69.5	65.3	58.2	52.6	49.2	42.8
Single parent	6.8	12.1	7.0	13.4	8.3	16.6
*Body Mass Index (%)*						
Normal or underweight	48.0	73.0				
Overweight	41.9	20.1				
Obese	10.1	6.9				
*Pack-years smoking (%)*						
0	40.0	58.8				
0.1–10	17.0	16.5				
10.1–20	16.5	9.6				
20.1–30	9.8	4.7				
> 30	9.8	2.6				
Missing	6.8	7.8				
*Leisure time physical activity (%)*						
None	46.4	48.2				
Light	18.6	26.0				
Regular	20.2	19.5				
Intense	14.7	6.3				
*Diabetes (%)*	2.3	1.6				
*Hypertension (%)*	9.6	8.9				
*Household economic resources (%)*						
Excellent or adequate	70.1	72.6				
Scarce or absolutely insufficient	29.9	27.4				
*Geographical area of residence (%)*						
North–West	22.2	24.5				
North–East	21.0	24.9				
Center	17.1	19.1				
South	39.7	31.5				

^1^Range: from -5.76 to 16.38

^2^Range: from -5.04 to 27.89

In ILS 2005, almost 43% men and 50% women were resident in the North of the country, followed by the South (40% men and 31% women) and the Center. More than half men were overweight or obese, whereas among women this proportion reached only one quarter. Forty per cent men and almost 60% women were never smokers, while about 20% men and 7% women had smoked more than 20 pack-years. In both genders almost half subjects did not perform any LTPA, and only 15% men and 6% women practiced intense LTPA. In both men and women, around one quarter reported that their household had scarce or absolutely insufficient economic resources. The prevalence of diabetes was 2% and that of hypertension less than 10% in both men and women.

Frequency distribution of subjects’ characteristics changed very little after excluding CVD prevalent cases, except for a slight decrease of the proportion of men and women with a Charlson index greater than zero, in TLS 2001 and 2011, and of those reporting hypertension in ILS 2005 (Supplementary Table 1).

### Occupational physical activity and mortality

#### ILS 2005

In the national cohort (ILS 2005), the analysis adjusted for age, household typology, PCS, economic resources, and area of residence, showed a significant association between mortality and continuous exposure to OPA (*p* = 0.02), with significantly higher mortality among men in the third (IRR = 1.42) and in the highest quartile of exposure to OPA (IRR = 1.37), compared to the lowest (Table 2, Model 1), and a significant trend with increasing exposure (*p* = 0.02). Adjustment also for educational level produced a slightly attenuation of the associations (Table 2, Model 2), which however lost significance. Adding to the model also behavioral (smoking, BMI, LTPA) and biological CVD risk factors (hypertension, diabetes) increased slightly the associations with mortality for the third and the fourth quartiles, which nonetheless remained non-significant (Model 3). In contrast, among women there was a weak inverse relationship between OPA quartile and mortality, which however did not reach significance in any model (Table 3). The analysis stratified by LTPA did not show in men or women relevant differences in the association between OPA and mortality in subjects performing or not LTPA (all *p* values for interaction > 0.05) (Supplementary Table 2).

**Table 2:** Incidence rate ratio (IRR) (and 95% CI) of mortality by quartile of Occupational Physical Activity (OPA). Poisson regression models for men

Mortality for men	ILS 2005			TLS 2001		TLS 2011	
	Model1^1^	Model2^2^	Model3^3^	Model1^4^	Model2^5^	Model1^4^	Model2^5^
	*β*	*β*	*β*	*β*	*β*	*β*	*β*
*Ergo-index* (continuous)	0.009*	0.007	0.009	0.010**	0.003	1.02*e *− 06**	9.48*e *− 07**
	**IRR (95% CI)**	**IRR (95% CI)**	**IRR (95% CI)**	**IRR (95% CI)**	**IRR (95% CI)**	**IRR (95% CI)**	**IRR (95% CI)**
*Ergo-index* (ref: 1° quartile)	1	1	1	1	1	1	1
2° quartile	1.13 (0.78–1.63)	1.10 (0.74–1.64)	1.08 (0.71–1.64)	1.33** (1.19–1.48)	1.13* (1.01–1.27)	0.97 (0.78–1.19)	0.97 (0.79–1.20)
3° quartile	1.42* (1.02–1.81)	1.37 (0.95–1.99)	1.42 (0.97–2.09)	1.30** (1.17–1.44)	1.04 (0.93–1.17)	1.13 (0.93–1.36)	1.12 (0.93–1.35)
4° quartile	1.37* (1.03–1.82)	1.31 (0.93–1.87)	1.36 (0.94–1.97)	1.43** (1.30–1.56)	1.11* (1.00–1.23)	1.32** (1.11–1.57)	1.27** (1.06–1.51)
*Age* (continuous)	1.10**	1.10**	1.08**	1.10**	1.10**	1.09**	1.08**
*Household typology* (ref: single)	1	1	1	1	1	1	1
Couple without children	0.75	0.75	0.75	0.78**	0.77**	0.76**	0.75**
Couple with children	0.77	0.77	0.77	0.70**	0.68**	0.67**	0.66**
Single parent	1.57*	1.57*	1.60*	1.00	0.98	1.04	1.03
*Charlson index* (continuous)				1.45**	1.46**	1.56**	1.56**
*Area deprivation index* (continuous)				1.05**	1.04**	1.06**	1.04**
*Physical Component Summary* (continuous)	0.96**	0.97**	0.97**				
*Household economic resources* (ref: excellent or adequate)	1	1	1				
Scarce or absolutely insufficient	1.50**	1.50**	1.42**				
*Geographical area of residence* (ref: North-West)	1	1	1				
North-East	1.26	1.26	1.23				
Center	1.14	1.14	1.06				
South	1.07	1.08	1.00				
Educational level (ref. university degree)		1	1		1		1
High school diploma		0.92	0.90		1.31**		1.25*
Low secondary or elementary		1.01	0.96		1.73**		1.38**
*Body Mass Index* (ref: normal or underweight)			1				
Overweight			0.89				
Obese			1.15				
*Pack-years smoking* (ref: 0 pack-years)			1				
0.1–10			1.20				
10.1–20			1.72**				
20.1–30			1.97**				
> 30			2.72**				
*Leisure time physical activity* (ref: no activity)			1				
Light			0.86				
Regular			1.32				
Intense			1.27				
*Diabetes*			1.07				
*Hypertension*			1.17				

^*^p < 0.05, **p < 0.01

^1^Adjusted for age, household typology, household economic resources, Physical Component Summary, geographical area of residence

^2^Adjusted for age, household typology, household economic resources, Physical Component Summary, geographical area of residence, educational level

^3^Adjusted for age, household typology, household economic resources, Physical Component Summary, geographical area of residence, educational level, BMI, pack-years smoking, leisure time physical activity, diabetes, hypertension

^4^Adjusted for age, household typology, Charlson index, area deprivation index

^5^Adjusted for age, household typology, Charlson index, area deprivation index, educational level

**Table 3:** Incidence rate ratio (IRR 95% CI) of mortality by quartile of Occupational Physical Activity (OPA). Poisson regression models for women

Mortality for women	ILS 2005			TLS 2001		TLS 2011	
	Model1^1^	Model2^2^	Model3^3^	Model1^4^	Model2^5^	Model1^4^	Model2^5^
	*β*	*β*	*β*	*β*	*β*	*β*	*β*
*Ergo-index* (continuous)	-0.003	-0.006	-0.007	0.005*	0.003	6.33e-07**	6.43e-07**
	**IRR (95% CI)**	**IRR (95% CI)**	**IRR (95% CI)**	**IRR (95% CI)**	**IRR (95% CI)**	**IRR (95% CI)**	**IRR (95% CI)**
*Ergo-index* (ref: 1° quartile)	1	1	1	1	1	1	1
2° quartile	0.67 (0.39–1.16)	0.63 (0.36–1.11)	0.56 (0.31–1.01)	1.12 (0.98–1.30)	1.09 (0.94–1.26)	1.30* (1.02–1.64)	1.29* (1.02–1.64)
3° quartile	0.84 (0.58–1.23)	0.76 (0.50–1.15)	0.76 (0.50–1.17)	1.05 (0.92–1.18)	0.98 (0.86–1.12)	1.52** (1.20–1.91)	1.52** (1.20–1.92)
4° quartile	0.87 (0.59–1.29)	0.78 (0.50–1.22)	0.74 (0.47–1.17)	1.28** (1.11–1.48)	1.20* (1.03–1.40)	1.71** (1.34–2.20)	1.73** (1.35–2.22)
*Age* (continuous)	1.10**	1.09**	1.09**	1.09**	1.09**	1.08**	1.08**
*Household typology* (ref: single)	1	1	1	1	1	1	1
Couple without children	0.60	0.59	0.67	0.86	0.85*	0.91	0.91
Couple with children	0.64*	0.63*	0.70	0.67**	0.66**	0.92	0.92
Single parent	0.70	0.69	0.74	1.07	1.05	1.23	1.23
*Charlson index* (continuous)				1.77**	1.78**	1.68**	1.69**
*Area deprivation index* (continuous)				1.05**	1.04**	1.04*	1.05*
*Physical Component Summary* (continuous)	0.95**	0.95**	0.95**				
*Household economic resources* (ref: excellent or adequate)	1	1	1				
Scarce or absolutely insufficient	1.17	1.15	1.12				
*Geographical area of residence* (ref: North–West)	1	1	1				
North–East	0.90	0.89	0.80				
Center	0.97	0.99	0.82				
South	0.98	1.00	0.91				
*Educational level* (ref. university degree)		1	1		1		1
High school diploma		1.10	0.99		1.18*		0.99
Low secondary or elementary		1.29	1.15		1.26**		0.89
*Body Mass Index* (ref: normal or underweight)			1				
Overweight			0.88				
Obese			1.14				
*Pack-years smoking* (ref: 0 pack-years)			1				
0.1–10			1.06				
10.1–20			1.11				
20.1–30			1.38				
> 30			3.02**				
*Leisure time physical activity* (ref: no activity)			1				
Light			0.44				
Regular			0.75				
Intense			0.71				
*Diabetes*			1.60				
*Hypertension*			0.92				

^*^p < 0.05, **p < 0.01

^1^Adjusted for age, household typology, household economic resources, Physical Component Summary, geographical area of residence

^2^Adjusted for age, household typology, household economic resources, Physical Component Summary, geographical area of residence, Educational level

^3^Adjusted for age, household typology, household economic resources, Physical Component Summary, geographical area of residence, educational level, BMI, pack-years smoking, leisure time physical activity, diabetes, hypertension

^4^Adjusted for age, household typology, Charlson index, area deprivation index

^5^Adjusted for age, household typology, Charlson index, area deprivation index, educational level

#### TLS 2001

In the TLS 2001 cohort, in the analysis adjusted for socio-demographics and health, but not for education (Table 2, Model I), in both genders mortality was significantly associated with continuous exposure to OPA, with risks increased by about 30–40% among men in all exposure quartiles, compared to the lowest, and with a significant trend in risk across increasing exposure quartiles (*p* value for trend < 0.001). In contrast, among women only the risk in the highest exposure quartile was significantly increased (IRR = 1.28), although also with a significant trend in risk with increasing exposure (*p* < 0.01) (Table 3, Model 1). Adjusting also for education, among women the association with the highest exposure quartile persisted (IRR = 1.20) (Table 3, Model 2), while among men all the associations more than halved, but remained significantly elevated in the second and the fourth quartiles (IRR = 1.13 and IRR = 1.11, respectively) (Table 2, Model 2).

#### TSL 2011

Regarding the TLS 2011 cohort, in both genders mortality was significantly associated with continuous exposure to OPA, with risks significantly increased in the highest cumulative exposure quartile (men: IRR = 1.32; women: IRR = 1.71), compared to the lowest, and also in the 2nd and the 3rd quartile among women (IRR = 1.30 and IRR = 1.52, respectively) (Table 2 and 3, Model 1), and with a significant trend in risk in both men and women (*p* value for trend < 0.01 for both). In the analysis adjusted also for education, these associations persisted, with only slight changes in IRRs in men or women (Table 2 and 3, Model 2).

### Occupational physical activity and CVD incidence

#### ILS 2005

In ILS 2005, exposure to OPA treated as a continuous variable was not associated with CVD among men in any model, whereas among women it showed in all models a significant association. In the analysis not adjusted for education (Tables 4 and 5, Model 1), men and women with higher cumulative exposure were at higher risk of developing CVD, with significant associations among men in the second and the third quartile (IRR = 1.25 for both) and among women in the third and the fourth quartile (IRR = 1.33 and 1.29, respectively), and with a significant trend in risk in women (*p* < 0.01), but not in men (*p* = 0.17). Further adjustment for education produced only a slight decrease of these associations, which remained all statistically significant (Tables 4 and 5, Model 2). Adding to the model also behavioural CVD risk factors, diabetes and hypertension, produced a slight decrease of the IRRs in the second exposure quartile among men and in the highest quartile among women, which lost significance (*p* = 0.08 and *p* = 0.11, respectively) (Tables 4 and 5, Model 3). As for mortality, in both genders the association of OPA with CVD incidence did not differ between subjects performing or not LTPA (all *p* values for interaction > 0.05) (Supplementary Table 2).

**Table 4:** Incidence rate ratio (IRR 95% CI) of cardiovascular diseases (CVD) by quartile of Occupational Physical Activity (OPA). Poisson regression models for Men

CVD for men	ILS 2005			TLS 2001		TLS 2011	
	Model1^1^	Model2^2^	Model3^3^	Model1^4^	Model2^5^	Model1^4^	Model2^5^
	Beta	Beta	Beta	Beta	Beta	Beta	Beta
*Ergo-index* (continuous)	0.002	0.0005	0.0007	0.004**	-0.001	3.06e-07	2.65e-08
	**IRR (95% CI)**	**IRR (95% CI)**	**IRR (95% CI)**	**IRR (95% CI)**	**IRR (95% CI)**	I**RR (95% CI)**	**IRR (95% CI)**
*Ergo-index* (ref: 1° quartile)	1	1	1	1	1	1	1
2° quartile	1.25** (1.06–1.48)	1.22* (1.02–1.45)	1.18 (0.98–1.41)	1.13** (1.05–1.20)	1.02 (0.95–1.09)	1.10 (0.97–1.24)	1.10 (0.98–1.25)
3° quartile	1.25** (1.07–1.46)	1.19* (1.00–1.42)	1.22* (1.02–1.46)	1.19** (1.12–1.26)	1.02 (0.95–1.09)	1.06 (0.94–1.19)	1.05 (0.93–1.18)
4° quartile	1.11 (0.97–1.28)	1.06 (0.90–1.25)	1.06 (0.89–1.26)	1.18** (1.12–1.25)	1.00 (0.93–1.06)	1.13* (1.01–1.26)	1.08 (0.97–1.20)
*Age* (continuous)	1.06**	1.06**	1.04**	1.05**	1.05**	1.07**	1.07**
*Household typology* (ref: single)	1	1	1	1	1	1	1
Couple without children	1.03	1.03	0.98	1.06	1.05	0.96	0.95
Couple with children	0.87	0.86	0.85	1.04	1.02	0.91*	0.90*
Single parent	1.00	0.99	0.98	1.02	1.00	1.03	1.01
*Charlson index* (continuous)				1.19**	1.19**	1.24**	1.23**
*Area deprivation index* (continuous)				1.02**	1.01*	1.01	1.00
*Physical Component Summary* (continuous)	0.99**	0.99**	0.99				
*Household economic resources* (ref: excellent or adequate)	1	1	1				
Scarce or absolutely insufficient	0.97	0.97	0.95				
*Geographical area of residence* (ref: North-West)	1	1	1				
North-East	0.84*	0.84*	0.85				
Center	0.94	0.94	0.94				
South	1.16*	1.16	1.13				
*Educational level* (ref. university degree)		1	1		1		1
High school diploma		1.23*	1.18		1.16**		1.17**
Low secondary or elementary		1.21	1.14		1.43**		1.29**
*Body Mass Index* (ref: normal or underweight)			1				
Overweight			1.07				
Obese			1.28**				
*Pack-years smoking* (ref: 0 pack-years)			1				
0.1—10			1.01				
10.1—20			1.05				
20.1—30			1.24*				
> 30			1.58**				
*Leisure time physical activity* (ref: no activity)			1				
Light			0.95				
Regular			0.99				
Intense			0.93				
*Diabetes*			1.58**				
*Hypertension*			1.36**				

^*^p < 0.05, **p < 0.01

^1^Adjusted for age, household typology, household economic resources, Physical Component Summary, geographical area of residence

^2^Adjusted for age, household typology, household economic resources, Physical Component Summary, geographical area of residence, Educational level

^3^Adjusted for age, household typology, household economic resources, Physical Component Summary, geographical area of residence, educational level, BMI, pack-years smoking, leisure time physical activity, diabetes, hypertension

^4^Adjusted for age, household typology, Charlson index, area deprivation index

^5^Adjusted for age, household typology, Charlson index, area deprivation index, educational level

**Table 5:** Incidence rate ratio (IRR 95% CI) of cardiovascular diseases (CVD) by quartile of Occupational Physical Activity (OPA). Poisson regression models for Women

CVD for women	ILS 2005			TLS 2001		TLS 2011	
	Model1^1^	Model2^2^	Model3^3^	Model1^4^	Model2^5^	Model1^4^	Model2^5^
	*β*	*β*	*β*	*β*	*β*	*β*	*β*
*Ergo-index* (continuous)	0.010**	0.008*	0.007*	0.016**	0.010**	4.68e-07**	4.21e-07*
	**IRR (95% CI)**	**IRR (95% CI)**	**IRR (95% CI)**	**IRR (95% CI)**	**IRR (95% CI)**	**IRR (95% CI)**	**IRR (95% CI)**
*Ergo-index* (ref: 1° quartile)	1	1	1	1	1	1	1
2° quartile	1.00 (0.79–1.28)	0.98 (0.77–1.26)	0.98 (0.76–1.26)	1.41** (1.30–1.52)	1.30** (1.20–1.41)	1.04 (0.92–1.17)	1.04 (0.92–1.17)
3° quartile	1.33** (1.13–1.57)	1.26* (1.04–1.53)	1.29** (1.06–1.57)	1.36** (1.27–1.45)	1.16** (1.07–1.24)	1.07 (0.95–1.20)	1.05 (0.93–1.19)
4° quartile	1.29** (1.08–1.55)	1.23* (1.00–1.52)	1.19 (0.96–1.47)	1.61** (1.49–1.74)	1.38** (1.27–1.49)	1.20** (1.05–1.37)	1.16* (1.02–1.33)
*Age* (continuous)	1.04**	1.04**	1.03**	1.03**	1.03**	1.05**	1.04**
*Household typology* (ref: single)	1	1	1	1	1	1	1
Couple without children	0.89	0.88	0.89	1.08	1.05	1.00	0.97
Couple with children	1.33*	1.31*	1.35*	1.16**	1.12**	1.13*	1.09
Single parent	1.30	1.29	1.31	1.15**	1.10*	1.16*	1.13
*Charlson index* (continuous)				1.19**	1.19**	1.19**	1.19**
*Area deprivation index* (continuous)				1.04**	1.03**	1.05**	1.03**
*Physical Component Summary* (continuous)	0.97**	0.98**	0.98**				
*Household economic resources* (ref: excellent or adequate)	1	1	1				
Scarce or absolutely insufficient	0.97	0.96	0.91				
*Geographical area of residence* (ref: North–West)	1	1	1				
North–East	1.00	1.01	1.03				
Center	1.08	1.08	1.04				
South	1.21	1.22*	1.18				
*Educational level* (ref. university degree)		1	1		1		1
High school diploma		1.25	1.26		1.12**		1.28**
Low secondary or elementary		1.24	1.19		1.50**		1.56**
*Body Mass Index* (ref: normal or underweight)			1				
Overweight			1.23*				
Obese			1.38**				
*Pack-years smoking* (ref: 0 pack-years)			1				
0.1–10			1.14				
10.1–20			1.25*				
20.1–30			1.44**				
> 30			1.75**				
*Leisure time physical activity* (ref: no activity)			1				
Light			0.93				
Regular			0.88				
Intense			0.86				
*Diabetes*			1.76**				
*Hypertension*			1.49**				

^*^p < 0.05, **p < 0.01

^1^Adjusted for age, household typology, household economic resources, Physical Component Summary, geographical area of residence

^2^Adjusted for age, household typology, household economic resources, Physical Component Summary, geographical area of residence, Educational level

^3^Adjusted for age, household typology, household economic resources, Physical Component Summary, geographical area of residence, educational level, BMI, pack-years smoking, leisure time physical activity, diabetes, hypertension

^4^Adjusted for age, household typology, Charlson index, area deprivation index

^5^Adjusted for age, household typology, Charlson index, area deprivation index, educational level

#### TLS 2001

In the TLS 2001 cohort, in the analysis not adjusted for education (Model 1), in both genders there was a significant association between exposure to OPA kept continuous and CVD risk, which persisted among women, but not among men, adding education to the model. The risk of CVD was significantly increased in all higher exposure quartiles, compared to the lowest, with stronger associations found among women (second quartile: IRR = 1.41, third quartile: IRR = 1.36, fourth quartile IRR = 1.61) than men (second quartile: IRR = 1.13, third quartile: IRR = 1.19, fourth quartile IRR = 1.18). Also, a significant trend in risk was estimated for both men and women (*p* value for trend < 0.01 for both). Further adjustment for educational level produced among men a complete disappearance of these associations (Model 2), whereas among women they decreased, compared to the previous analysis, but remained significant in all higher quartiles, compared to the lowest (second quartile: IRR = 1.30, third quartile: IRR = 1.16, fourth quartile IRR = 1.38).

#### TLS 2011

In the TLS 2011 cohort, among women exposure to continuous cumulative OPA was significantly associated with CVD risk in both models (adjusted or not for education), whereas among men no association was found in either model. Considering cumulative OPA in quartiles, results from Model 1 showed a significantly increased risk in both genders in the highest quartile of cumulative exposure to OPA (men: IRR = 1.13, women: IRR = 1.20), with a dose–response trend among both women (*p* < 0.01) and men (*p* < 0.05). Adjusting also for education (Model 2) produced in both genders a decrease of the associations, although lower than for TLS 2001, with the risk of CVD remaining significantly elevated only among women in the highest exposure quartile (IRR = 1.16).

In no higher OPA quartile the risk of mortality or CVD was significantly reduced among men or women, compared to the lowest quartile, in any of the cohorts.

##### Sensitivity analysis

Very similar results to those of the main analysis were found on mortality and CVD in TLS 2001 after exclusion of subjects present also in the TLS 2011 cohort (Supplementary Table 3).

## Discussion

### Main findings

In the present study, an increased risk of mortality associated with exposure to high OPA, assessed by means of a JEM, was found among men in all cohorts examined and among women in two of them, controlling for sociodemographic and health characteristics. These associations decreased, but remained significant or marginally significant further adjusting for education and other covariates. For CVD, the highest exposure quartile was at significantly higher risk in both men and women, adjusting for socio-demographics and health, while with further adjustment for education these associations lost significance among men, but not among women. In contrast, no association was found for LTPA with mortality or CVD incidence in men or women.

### Interpretation of the results

Our results indicate that high OPA does not have a beneficial effect on CVD or mortality, in contrast to the protective effects demonstrated for LTPA. Instead, the rather good consistency of our findings on mortality across cohorts and genders, gives support to the hypothesis that OPA has a detrimental effect on health, also considering that the analysis on mortality in the ILS 2005 cohort had relatively low statistical power, being estimated on a limited number of death, especially among women (*N* = 179 in total), and therefore provided risk estimates characterized by a wide uncertainty. In spite CVD incidence was not associated with OPA in any cohort in men, except for the third quartile in ILS 2005, the consistency of our results among women, also showing positive dose–response trends with higher OPA in all cohorts, seems to indicate the possibility of a negative effect of OPA on health, too. Furthermore, the stronger attenuation of the risk of CVD observed among men in both ILS 2005 and TLS 2001 after controlling for education should be interpreted with caution, as the strong correlations present between the Ergo-index and education could have caused, because of model multicollinearity, an artificial attenuation of the risk estimates, as well as an increase in their standard errors (Vatcheva et al. 2016).

A possible explanation for the lack of a beneficial effect from LTPA in the present study, in contrast with findings in the literature (Cheng et al. 2018), is that only activities performed at least once a week were considered, so that subjects performing LTPA less regularly were classified as inactive. This resulted in a high proportion of people not performing any physical activity (almost half of the study population), part of which have been likely misclassified, leading to an attenuation of the associations between LTPA and the outcomes. However, even higher proportion of physically inactive subjects have been reported for Italy in two EU Special Eurobarometers surveys, although they included also older subjects, whereas our study was restricted only to subjects of working age (Mayo et al. 2019).

The associations of exposure to OPA with mortality and CVD in the different cohorts were in the same direction and were quite comparable among men, also considering the large uncertainty characterizing many risk estimates. Among women, IRRs for CVD were also in the same direction and of similar strength in the three cohorts, although slightly higher in the TLS 2001 cohort. In contrast, IRRs for mortality among women were heterogeneous across cohorts, especially those for mortality between TLS 2011, where significant positive associations were found in all higher exposure quartiles, compared to the lowest, and ILS 2005, in which IRRs below 1 were estimated for all higher quartiles, although none of these associations was significant. It appears difficult to explain the discrepancy of the results on mortality among women with all others, although a possible explanation of such a heterogeneity is the different populations investigated, which in ILS 2005 was representative of the national population, while the TLS cohorts included the whole urban population of Turin, in both cases restricted to employees of working age. Turin is a town with more than 800,000 inhabitants, where women’s employment rate is higher than in the whole country (64.5% vs. 56.4% at 2011 census in the age class 15–64 years). Therefore, at the national level employed women may be subjected to stronger health selection than those in the urban cohorts (the “healthy worker effect”), especially those exposed to high OPA, which would lead to an underestimation of the association between OPA and mortality or CVD incidence. Also, the lower statistical power of the analyses of ILS 2005 may have contributed to a reduced number of significant findings in this cohort, compared to the other two.

The results of the sensitivity analysis on the TLS 2001 cohort, which excluded subjects present also in the TSL 2011 cohort, showed very similar results to those of the main analysis, indicating that the relative consistency of the results between the two TLS cohort was not inflated by the presence of overlapping subjects.

Differences in the type of activity performed in OPA and LTPA, especially in terms of intensity, duration and recovery pauses, have been suggested as a possible explanation of the contrasting effects of OPA and LTPA on health and mortality. In fact, OPA would rarely be of such high intensity to improve cardiorespiratory fitness, in contrast with LTPA where efforts corresponding to 60–80% of the maximal aerobic capacity are reached; furthermore, OPA is performed for many hours with short pauses, whereas LTPA is generally performed for short periods of time (Holtermann et al. 2018). Such low-intensity physical activity, which involves more static muscle contraction than LTPA and is sustained for prolonged time with insufficient recovery, would cause an imbalance in the autonomic regulation, with a consequent predominance of the sympathetic over the parasympathetic system (Hallman et al. 2017), which has been proposed as an underlying mechanism of the effect of OPA on the risk of CVD and mortality (Thayer et al. 2010). The increased activity of the sympathetic system would lead to permanent increases in heart rate and blood pressure (Clays et al. 2012), which in turn would increase the risk of vessels inflammation (Lee et al. 2021; Feinberg et al. 2022) and of development of atherosclerotic processes in the arteries (Ujka et al. 2017; Krause et al. 2007; Korshoj et al. 2023).

### Comparison with other studies

Regarding mortality, for men our results appear quite consistent with those of the meta-analysis by Coenen et al. (2018), where an 18% increased risk was estimated, and with some more recent studies. Among them, Holtermann et al. (2021) also found increased mortality associated with high OPA, with relative risks comparable to ours, although results were not presented by gender; in this study risks almost halved adjusting for lifestyles, education, health and biological risk factors, but the association persisted for exposure to high or very high OPA. A Finnish study estimated a 14% increased risk of mortality among workers exposed to heavy manual labour, compared to those reporting mostly walking and lifting, in an analysis controlled for a similar set of covariates as above (Hermansen et al. 2019). Furthermore, an analysis of two Swiss cohorts found in one of them a significantly increased risk of mortality (25%), although no association was found in the other one (Wanner et al. 2019). A significant increase in mortality was also found in a US study, but risks strongly decreased controlling for socioeconomic characteristics, lifestyles and health, remaining slightly increased only in men with exposure longer than 10 years (Martinez-Gomez et al. 2022). In contrast, Dalene et al. (2021) found a significant increase in longevity among men by almost two years for exposure to heavy physical work, in an analysis adjusted for several potential confounders, including education and LTPA. Our results for women in TLS 2001 and 2011 cohorts appear instead at odds with those of most studies, where no association was found (Coenen et al. 2018; Wanner et al. 2019; Dalene et al. 2021; Martinez-Gomez et al. 2022). Other two studies on mortality did not find any association in men or women (Luo et al. 2022; Pearce et al. 2021), but their definition of OPA was based on performing manual work or/and walking/standing, which may not adequately capture the high physical workload dimension of OPA.

For CVDs, evidence of an association with OPA in men or women appear more controversial, also because fewer studies are available on this issue. The most recent meta-analysis on OPA and CVD mortality did not find any association, but a non-significant 9% excess risk was estimated when considering only coronary heart disease mortality (Cillekens et al. 2023). However, the results of this meta-analysis are only in part generalizable to CVD incidence, as mortality may be influenced by several other factors in those affected, like comorbidities, access to health care and lifestyles. Among studies which assessed the relation of OPA with CVD incidence, rather than CVD mortality, the study by Holtermann et al. (2021) found an increased risk for exposure to high and very high OPA, which persisted after adjustment for many potential confounders. Also, an increased risk of stroke by almost 50% was observed in a cohort of US women for exposure to high OPA in the longest held job, which decreased only slightly in fully adjusted models (Hall et al. 2019). Another US study, based on the National Health Interview Survey data, found a doubling in CVD risk, with a dose–response trend, associated with frequent occupational exertion in both genders, after adjusting for lifestyles, socioeconomic characteristics and health (Quinn et al. 2021). However, no association of high OPA with coronary heart disease was found in another cohort of women in the US, even in models with minimal adjustment for socioeconomic and CVD risk factors (Wang et al. 2019), and with CVD in a Dutch cohort of both genders (Bonekamp et al. 2022).

Last, the results of the analyses do not support an effect modification by LTPA on the association of OPA with mortality or CVD risk, in contrast with the findings of different studies, where the association was stronger for subjects with a low level of LTPA (Wang et al. 2019; Clays et al. 2013; Harari et al. 2015; Prince et al. 2021). The substantial misclassification of exposure to LTPA expected in the ILS 2005 cohort, discussed above, may have led to underestimate both the associations of LTPA with mortality and CVD, as well as effect modification by LTPA on the association between OPA and these health outcomes.

### Strengths

Main strength of this study consists in the large cohorts enrolled, although the restriction of the study populations to subjects relatively young limited the statistical power of the analyses, preventing the possibility to examine with sufficient statistical power the relationship between OPA and mortality in the ILS cohort, which was one-fifth in size of the two other cohorts. On the other hand, the restriction to workers below statutory pension age at baseline is itself a strength of the study, as it reduced the possibility of selection of healthier older workers into the cohorts, in particular those exposed to high OPA (i.e., the “healthy worker effect”), which could have caused an underestimation of the true association between OPA and the health outcomes. It seems possible that the results of several negative studies including relevant proportions of older subjects may have been affected by such a bias (Dalene et al. 2021; Wang et al. 2019; Bonekamp et al. 2022; Huerta et al. 2016; Luo et al. 2022; Pearce et al. 2021).

Another strength is that both exposure to OPA and occurrence of the health outcomes were assessed using the same methodology in all three different datasets, which allowed to compare meaningfully the results of the three cohorts and evaluate their consistency, in spite of differences in the available information on covariates and in territorial and temporal coverage between the cohorts.

Exposure to OPA was assigned through a JEM, which has among its strengths the possibility to attribute to a working population exposure to hazards at work in a standardized and reproducible way, based on job title, industry, and, in some cases, period of exposure. Furthermore, the use of JEM prevents the possibility of exposure over-reporting by less healthy subjects, which could have caused spurious positive associations between the exposure and the outcomes. A study comparing self-reported exposure to OPA with that obtained through objective worn-device measurements actually found that self-reported assessment overestimated workload by 51%, compared to objective measures, with a significantly higher overestimate for self-reports among workers with low cardiorespiratory fitness, confirming such a possibility (Korshoj et al. 2022). On the other hand, as JEM ignore inter-individual variability within jobs, all workers holding the same job title are assigned the same exposure value; this may increase non-differential exposure misclassification, depending on the JEM specificity, which would cause an attenuation of the true exposure-outcome associations. However, the only two previous studies in which exposure to OPA was assigned through a JEM, also found significantly increased mortality associated with high OPA (Ervasti et al. 2019; Mikkola et al. 2019). Another problem with JEM, in particular for assessing exposures in occupations showing a strong social gradient like for physical workload, is that exposure scores may be strongly correlated with socioeconomic status, as found in the present study and also in Swedish (d’Errico et al. 2022b) and Finnish data (Mikkola et al.^2019^), because they are assigned as mean values to a job, without considering intra-job exposure variability. In these situations, a substantial attenuation of the associations between OPA and health outcomes and a distortion of their confidence intervals is expected, as discussed above.

Furthermore, the Ergo-index of the JEM, which was our measure of OPA, was constructed from many different ergonomic exposures, so that it reflects different dimensions of physical workload, including also awkward postures and repetitiveness, besides high physical effort. These other dimensions may be also relevant in increasing the risk of mortality or CVD, as suggested by the fact that in a previous study on the ILS cohort, in which OPA was assessed through self-reports at baseline based on one question on intensity of physical activity at work, no association was found between high OPA and mortality or coronary heart disease incidence in neither gender (Strippoli et al. 2022). It is worth noting that in several other studies reporting no association, exposure assessment to OPA was also rather crude, as it was mainly based on one question on the type of job performed (e.g. sedentary, walking, heavy effort, etc.) at one or two points in time, which implies a substantial misclassification of true cumulative exposure to OPA during working life (Huerta et al. 2016; Menotti et al. 2016; Wanner et al. 2014; Petersen et al. 2012; Luo et al. 2022; Pearce et al. 2021). The stronger associations of high OPA with mortality observed in both genders in the TLS 2011, in which exposure was assigned based on jobs held along many years, compared to TLS 2001, where it was based on only the job held at baseline, suggest how crucial is the accurate reconstruction of cumulative exposure to OPA to correctly assess its impact on health. Similarly, in the two studies on mortality where OPA was assessed through a JEM, higher risks were observed in the study by Ervasti et al. (2019), in which exposure was computed as the average exposure in the last 10 years, compared to the study by Mikkola et al. (2019), where it was limited to the job held at baseline.

### Limitations

While the ILS 2005 cohort was a representative sample of the national population and the TLS 2001 cohort included the whole Turin population of employees of working age, the TLS 2011 cohort was not a representative sample of the residents working as employees at 2011 census. In fact, in this cohort were selected only subjects with job contracts starting or ending from 2009 to 2018, therefore workers with shorter length of contracts were likely overrepresented. However, it should be noted that workers with long careers, but with at least one discontinuity or termination of the employment relationship, or retirement, have in any case been selected in the study, also considering that the labour market has become more and more flexible in the last years, especially for young people, but also for older workers at the end of their career. The frequency distribution of educational level was quite comparable across the three cohorts, indicating that the cohorts had a similar socioeconomic structure. A comparison with data from 2011 census reveals in the TLS 2011 cohort slightly higher proportions of subjects with low education, fixed-term contracts, low-skilled and unskilled workers, as well as some differences in the economic sectors of employment, compared to workers not included in the study (data not shown).

Although our results for the TLS 2001 and TLS 2011 cohorts may have been affected by confounding by behavioural factors, the adjustment performed for both educational level and a census tract deprivation index, as well as for health status, is expected to have contributed to control for confounding by these covariates, due to the presence of a socioeconomic gradient in the prevalence of exposure to such factors in Italy (Petrelli et al. 2022), as in other European countries (Mäki et al. 2014). Furthermore, adjustment for smoking, BMI, and LTPA produced only a small attenuation of the associations of OPA with mortality and CVD in the ILS 2005 cohort, suggesting that such factors are unlikely important confounders of the association between OPA and health.

## Conclusion

In the present study, we found an increased risk of mortality associated with exposure to high OPA in all cohorts among men and in two of three among women, adjusting for potential confounders. Also for CVD, both men and women in the highest exposure quartile were at significantly higher risk, although adjusting for education these associations persisted only in women.

Our results indicate that OPA does not reduce the risk of mortality or CVD, differently from LTPA, but rather support the “physical activity paradox” hypothesis that high OPA may be harmful for health. Preventive workplace interventions should reduce the intensity of physical effort, also limiting the duration of sustained physical work through an increase of recovery periods.

### Supplementary Information

Below is the link to the electronic supplementary material.

Supplementary file1 (DOCX 73 KB)

## Data Availability

Data used for the analyses are subjected to the legal restrictions established by the European privacy law, as the data contain potentially identifying or sensitive patient information. Open access to data is not possible but collaborations in specific projects with other research groups or institutes are possible upon collaboration agreement approval from the Presidential Committee of the Italian National Institute of Statistics.

## References

[CR1] Apolone G, Mosconi P, Quattrociocchi L, Gianicolo E, Groth N, Ware Jr JE. Questionario sullo stato di salute SF-12 [Health Status Questionnaire SF-12]. Milano, Italia: Istituto di Ricerche farmacologiche Mario Negri, 2001. Book in Italian.

[CR2] Bonekamp NE, Visseren FLJ, Ruigrok Y, Cramer MJM, de Borst GJ, May AM, Koopal C, UCC-SMART Study group; UCC-SMART study group (2023). Leisure-time and occupational physical activity and health outcomes in cardiovascular disease. Heart.

[CR3] Bull FC, Al-Ansari SS, Biddle S (2020). World Health Organization 2020 guidelines on physical activity and sedentary behaviour. Br J Sports Med.

[CR4] Cheng W, Zhang Z, Cheng W, Yang C, Diao L, Liu W (2018). Associations of leisure-time physical activity with cardiovascular mortality: a systematic review and meta-analysis of 44 prospective cohort studies. Eur J Prev Cardiol.

[CR5] Cillekens B, Huysmans MA, Holtermann A, van Mechelen W, Straker L, Krause N, van der Beek AJ, Coenen P (2022) Re: Cillekens B, Huysmans MA, Holtermann A, van Mechelen W, Straker L, Krause N, van der Beek AJ, Coenen P (2023) Physical activity at work may not be health enhancing A systematic review with meta-analysis on the association between occupational physical activity and cardiovascular disease mortality covering 23 studies with 655 892 participants. Scand J Work Environ Health 48(2):86–98. 10.5271/sjweh.3993. Scand J Work Environ Health 49(3):231–244. 10.5271/sjweh.4090 10.5271/sjweh.3993 PMC9045238 34656067

[CR6] Clays E, De Bacquer D, Van Herck K, De Backer G, Kittel F, Holtermann A (2012). Occupational and leisure time physical activity in contrasting relation to ambulatory blood pressure. BMC Public Health.

[CR7] Clays E, De Bacquer D, Janssens H, De Clercq B, Casini A, Braeckman L, Kittel F, De Backer G, Holtermann A (2013). The association between leisure time physical activity and coronary heart disease among men with different physical work demands: a prospective cohort study. Eur J Epidemiol.

[CR8] Coenen P, Huysmans MA, Holtermann A, Krause N, van Mechelen W, Straker LM, van der Beek AJ (2018). Do highly physically active workers die early? A systematic review with meta-analysis of data from 193 696 participants. Br J Sports Med.

[CR9] Coenen P, Huysmans MA, Holtermann A, Krause N, van Mechelen W, Straker LM, van der Beek AJ (2020). Towards a better understanding of the 'physical activity paradox': the need for a research agenda. Br J Sports Med.

[CR10] Dalene KE, Tarp J, Selmer RM, Ariansen IKH, Nystad W, Coenen P, Anderssen SA, Steene-Johannessen J, Ekelund U (2021). Occupational physical activity and longevity in working men and women in Norway: a prospective cohort study. Lancet Public Health.

[CR11] d'Errico A, Gallo F, Evanoff BA, Descatha A, Dale AM (2022). Reliability of O*NET physical exposures between Italian and US databases. Am J Ind Med.

[CR12] d'Errico A, Falkstedt D, Almroth M, Badarin K, Hemmingsson T, Kjellberg K (2022). Long-term sick leave for back pain, exposure to physical workload and psychosocial factors at work, and risk of disability and early-age retirement among aged Swedish workers. Int Arch Occup Environ Health.

[CR13] d'Errico A, Fontana D, Sebastiani G, Ardito C (2023). Risk of symptomatic osteoarthritis associated with exposure to ergonomic factors at work in a nationwide Italian survey. Int Arch Occup Environ Health.

[CR14] Ervasti J, Pietiläinen O, Rahkonen O, Lahelma E, Kouvonen A, Lallukka T, Mänty M (2019). Long-term exposure to heavy physical work, disability pension due to musculoskeletal disorders and all-cause mortality: 20-year follow-up-introducing Helsinki Health Study job exposure matrix. Int Arch Occup Environ Health.

[CR15] Flower DJC, Tipton MJ, Milligan GS (2019). Considerations for physical employment standards in the aging workforce. Work.

[CR16] Fontana D, Ardito C, Costa G, Boschetto B, d’Errico A (2022). Predictive validity of an indicator of exposure to unfavorable ergonomic working conditions on work-related musculoskeletal disorders. Qual Quant.

[CR17] Green F, McIntosh S (2001). The intensification of work in Europe. Labour Econ.

[CR18] Hall C, Heck JE, Sandler DP, Ritz B, Chen H, Krause N (2019). Occupational and leisure-time physical activity differentially predict 6-year incidence of stroke and transient ischemic attack in women. Scand J Work Environ Health.

[CR19] Hallman DM, Holtermann A, Søgaard K, Krustrup P, Kristiansen J, Korshøj M (2017). Effect of an aerobic exercise intervention on cardiac autonomic regulation: a worksite RCT among cleaners. Physiol Behav.

[CR20] Harari G, Green MS, Zelber-Sagi S (2015). Combined association of occupational and leisure-time physical activity with all-cause and coronary heart disease mortality among a cohort of men followed-up for 22 years. Occup Environ Med.

[CR21] Hermansen R, Jacobsen BK, Løchen ML, Morseth B (2019). Leisure time and occupational physical activity, resting heart rate and mortality in the Arctic region of Norway: the Finnmark Study. Eur J Prev Cardiol.

[CR22] Holtermann A, Krause N, van der Beek AJ, Straker L (2018). The physical activity paradox: six reasons why occupational physical activity (OPA) does not confer the cardiovascular health benefits that leisure time physical activity does. Br J Sports Med.

[CR23] Holtermann A, Schnohr P, Nordestgaard BG, Marott JL (2021). The physical activity paradox in cardiovascular disease and all-cause mortality: the contemporary Copenhagen General Population Study with 104,046 adults. Eur Heart J.

[CR24] Huerta JM, Chirlaque MD, Tormo MJ, Buckland G, Ardanaz E, Arriola L, Gavrila D, Salmerón D, Cirera L, Carpe B, Molina-Montes E, Chamosa S, Travier N, Quirós JR, Barricarte A, Agudo A, Sánchez MJ, Navarro C (2016). Work, household, and leisure-time physical activity and risk of mortality in the EPIC-Spain cohort. Prev Med.

[CR25] Kagan AR (1976). Atherosclerosis and myocardial disease in relation to physical activity of occupation. Bull World Health Organ.

[CR26] Korshøj M, Gupta N, Mortensen OS, Jørgensen MB, Holtermann A (2022). Intensity of occupational physical activity in blue-collar workers: do self-reported rating and device-worn measurements agree?. Eur J Appl Physiol.

[CR27] Korshøj M, Allesøe K, Mortensen OS, Siersma V, Kauhanen J, Krause N (2023). Occupational physical activity predicts baseline and 8-year progression of carotid atherosclerosis among women. Scand J Med Sci Sports.

[CR28] Krause N, Brand RJ, Kaplan GA, Kauhanen J, Malla S, Tuomainen TP, Salonen JT (2007). Occupational physical activity, energy expenditure and 11-year progression of carotid atherosclerosis. Scand J Work Environ Health.

[CR29] Lee J, Kim HR, Jang TW, Lee DW, Lee YM, Kang MY (2021). Occupational physical activity, not leisure-time physical activity, is associated with increased high-sensitivity C reactive protein levels. Occup Environ Med.

[CR30] Li J, Loerbroks A, Angerer P (2013). Physical activity and risk of cardiovascular disease: what does the new epidemiological evidence show?. Curr Opin Cardiol.

[CR31] Luo M, Gupta N, Holtermann A, Stamatakis E, Ding D (2022). Revisiting the 'physical activity paradox' in a Chinese context: occupational physical activity and mortality in 142,302 urban working adults from the China Kadoorie Biobank study. Lancet Reg Health West Pac.

[CR32] Mäki NE, Martikainen PT, Eikemo T, Menvielle G, Lundberg O, Ostergren O, Mackenbach JP, EURO-GBD-SE consortium members (2014). The potential for reducing differences in life expectancy between educational groups in five European countries: the effects of obesity, physical inactivity and smoking. J Epidemiol Community Health.

[CR33] Martinez Gomez D, Coenen P, Celis-Morales C, Mota J, Rodriguez-Artalejo F, Matthews C, Saint-Maurice PF (2022). Lifetime high occupational physical activity and total and cause-specific mortality among 320 000 adults in the NIH-AARP study: a cohort study. Occup Environ Med.

[CR34] Mayo X, Liguori G, Iglesias-Soler E, Copeland RJ, Clavel San Emeterio I, Lowe A, Del Villar F, Jimenez A (2019). The active living gender's gap challenge: 2013–2017 Eurobarometers physical inactivity data show constant higher prevalence in women with no progress towards global reduction goals. BMC Public Health.

[CR35] Menotti A, Puddu PE, Maiani G, Catasta G (2016). Cardiovascular and other causes of death as a function of lifestyle habits in a quasi extinct middle-aged male population. A 50-year follow-up study. Int J Cardiol.

[CR36] Mikkola TM, von Bonsdorff MB, Salonen MK, Kautiainen H, Ala-Mursula L, Solovieva S, Viikari-Juntura E, Eriksson JG (2019). Physical heaviness of work and sitting at work as predictors of mortality: a 26-year follow-up of the Helsinki Birth Cohort Study. BMJ Open.

[CR37] Morris JN, Heady JA, Raffle PA, Roberts CG, Parks JW (1953). Coronary heart-disease and physical activity of work. Lancet.

[CR38] Pearce M, Strain T, Wijndaele K, Sharp SJ, Mok A, Brage S (2021). Is occupational physical activity associated with mortality in UK Biobank?. Int J Behav Nutr Phys Act.

[CR39] Peters S (2020). Although a valuable method in occupational epidemiology, job-exposure-matrices are no magic fix. Scand J Work Environ Health.

[CR40] Petersen CB, Eriksen L, Tolstrup JS, Søgaard K, Grønbaek M, Holtermann A (2012). Occupational heavy lifting and risk of ischemic heart disease and all-cause mortality. BMC Public Health.

[CR41] Petrelli A, Sebastiani G, Di Napoli A, Macciotta A, Di Filippo P, Strippoli E, Mirisola C, d'Errico A (2022). Education inequalities in cardiovascular and coronary heart disease in Italy and the role of behavioral and biological risk factors. Nutr Metab Cardiovasc Dis.

[CR42] Prince SA, Rasmussen CL, Biswas A, Holtermann A, Aulakh T, Merucci K, Coenen P (2021). The effect of leisure time physical activity and sedentary behaviour on the health of workers with different occupational physical activity demands: a systematic review. Int J Behav Nutr Phys Act.

[CR43] Quan H, Sundararajan V, Halfon P, Fong A, Burnand B, Luthi JC, Saunders LD, Beck CA, Feasby TE, Ghali WA (2005). Coding algorithms for defining comorbidities in ICD-9-CM and ICD-10 administrative data. Med Care.

[CR44] Quinn TD, Yorio PL, Smith PM, Seo Y, Whitfield GP, Barone GB (2021). Occupational physical activity and cardiovascular disease in the United States. Occup Environ Med.

[CR45] Reinhardt JD, Wahrendorf M, Siegrist J (2013). Socioeconomic position, psychosocial work environment and disability in an ageing workforce: a longitudinal analysis of SHARE data from 11 European countries. Occup Environ Med.

[CR46] Rosano A, Pacelli B, Zengarini N, Costa G, Cislaghi C, Caranci N (2020). Aggiornamento e revisione dell’indice di deprivazione italiano 2011 a livello di sezione di censimento [Update and review of the 2011 Italian deprivation index calculated at the census section level]. Epidemiol Prev.

[CR47] Rose CL, Cohen ML (1977). Relative importance of physical activity for longevity. Ann N Y Acad Sci.

[CR48] Sebastiani G, Di Filippo P, Demaria M, Caranci N, Di Minco L, Tamburini C, d’Errico A, Grippo F, Costa G (2019) Lo studio longitudinale italiano: integrazione delle indagini sulla salute con dati di mortalità e ospedalizzazione. Metodologia e potenzialità di utilizzo [The Italian longitudinal study: integration of health surveys with mortality and hospitalization data. Methodology and potential of use.]. Istat. Article in Italian.

[CR49] Strippoli E, Hughes A, Sebastiani G, Di Filippo P, d'Errico A (2022). Occupational physical activity, mortality and CHD events in the Italian Longitudinal Study. Int Arch Occup Environ Health.

[CR50] Thayer JF, Yamamoto SS, Brosschot JF (2010). The relationship of autonomic imbalance, heart rate variability and cardiovascular disease risk factors. Int J Cardiol.

[CR51] Ujka K, Bruno RM, Bastiani L, Bernardi E, Sdringola P, Dikic N, Basyal B, Bhandari SS, Basnyat B, Cogo A, Pratali L (2017). Relationship between occupational physical activity and subclinical vascular damage in moderate-altitude dwellers. High Alt Med Biol.

[CR52] Vatcheva KP, Lee M, McCormick JB, Rahbar MH (2016). Multicollinearity in regression analyses conducted in epidemiologic studies. Epidemiology (sunnyvale).

[CR53] Wang C, De Roos AJ, Fujishiro K, Allison MA, Wallace R, Seguin RA, Nassir R, Michael YL (2019). Occupational physical activity and coronary heart disease in women's health initiative observational study. J Gerontol A Biol Sci Med Sci.

[CR54] Wanner M, Lohse T, Braun J, Cabaset S, Bopp M, Krause N, Rohrmann S, For The Swiss National Cohort Study Group (2019). Occupational physical activity and all-cause and cardiovascular disease mortality: results from two longitudinal studies in Switzerland. Am J Ind Med.

[CR55] Ware J, Kosinski M, Keller SD (1996). A 12-Item Short-Form Health Survey: construction of scales and preliminary tests of reliability and validity. Med Care.

