# Supplementary material for: Occupational physical activity, all-cause mortality and incidence of cardiovascular diseases: results from three Italian cohorts
Source: Int Arch Occup Environ Health. 2023 Dec 15;97(1):81–100. doi: 10.1007/s00420-023-02028-w (PMC10791782; doi:10.1007/s00420-023-02028-w)
Supplement: Supplementary file 1 — Supplementary file1 (DOCX 73 KB) [file 420_2023_2028_MOESM1_ESM.docx]

**Supplementary Table 1 – Frequency distribution of the characteristics of the three cohorts, CVD cumulative incidence and person-days of follow-up, by gender, after exclusion of CVD prevalent cases.**

| **Variables** | **ILS 2005** | | **TLS 2001** | | **TLS 2011** | |
| --- | --- | --- | --- | --- | --- | --- |
|  | ***Men*** | ***Women*** | ***Men*** | ***Women*** | ***Men*** | ***Women*** |
| ***Age at baseline (mean - sd)*** | 40.9 (9.1) | 40.9 (9.0) | 39.2 (8.6) | 39.0 (8.5) | 39.6 (9.6) | 39.5 (9.4) |
| ***Charlson index (% subjects >0)*** |  |  | 2.4 | 2.2 | 1.9 | 2.1 |
| ***Deprivation index. (mean - sd)*** |  |  | 0.11^1^ (2.0) | -0.08^1^ (1.8) | 1.64^2^ (2.1) | 1.48^2^ (2.0) |
| ***CVD cumulative incidence (%)*** | 9.3 | 5.8 | 12.3 | 8.3 | 4.3 | 2.6 |
| ***N. subjects*** | 15,307 | 15,423 | 75,994 | 76,322 | 70,970 | 77,287 |
| ***Person-days of follow-up (mean - sd)*** | 3,354.9 (621.4) | 3,414.5 (524.8) | 4,908.1 (1,990.8) | 5,174.3 (1,869.2) | 2,245.2 (95.0) | 2,272.8 (87.1) |
| ***Physical Component Summary (mean - sd)*** | 53.3 (6.3) | 52.4 (7.1) |  |  |  |  |
| ***Educational level (%)*** |  |  |  |  |  |  |
| University | 11.6 | 14.8 | 17.9 | 22.1 | 20.2 | 24.0 |
| High school | 30.8 | 34.8 | 32.7 | 35.6 | 30.1 | 32.9 |
| Low secondary/elementary | 57.6 | 50.4 | 49.3 | 42.3 | 49.7 | 43.1 |
| ***Household typology (%)*** |  |  |  |  |  |  |
| Single | 12.6 | 9.8 | 18.1 | 16.0 | 26.6 | 24.3 |
| Couple without children | 11.0 | 12.8 | 16.9 | 18.2 | 16.0 | 16.6 |
| Couple with children | 69.5 | 65.3 | 58.0 | 52.5 | 49.1 | 42.7 |
| Single parent | 6.9 | 12.0 | 6.9 | 13.3 | 8.3 | 16.4 |
| ***Body Mass Index (%)*** |  |  |  |  |  |  |
| Normal or underweight | 48.4 | 73.2 |  |  |  |  |
| Overweight | 41.8 | 20.0 |  |  |  |  |
| Obese | 9.8 | 6.8 |  |  |  |  |
| ***Pack-years smoking (%)*** |  |  |  |  |  |  |
| 0 | 40.4 | 58.9 |  |  |  |  |
| 0.1 - 10 | 17.1 | 16.5 |  |  |  |  |
| 10.1 - 20 | 16.5 | 9.6 |  |  |  |  |
| 20.1 - 30 | 9.7 | 4.7 |  |  |  |  |
| > 30 | 9.4 | 2.5 |  |  |  |  |
| Missing | 6.8 | 7.8 |  |  |  |  |
| ***Leisure time physical activity (%)*** |  |  |  |  |  |  |
| None | 46.4 | 48.2 |  |  |  |  |
| Light | 18.3 | 25.9 |  |  |  |  |
| Regular | 20.3 | 19.5 |  |  |  |  |
| Intense | 15.0 | 6.4 |  |  |  |  |
| ***Diabetes (%)*** | 2.0 | 1.4 |  |  |  |  |
| ***Hypertension (%)*** | 8.8 | 8.3 |  |  |  |  |
| ***Household economic resources (%)*** |  |  |  |  |  |  |
| Excellent or adequate | 70.3 | 72.7 |  |  |  |  |
| Scarce or absolutely insufficient | 26.7 | 27.3 |  |  |  |  |
| ***Geographical area of residence (%)*** |  |  |  |  |  |  |
| North-West | 22.1 | 24.6 |  |  |  |  |
| North-East | 21.0 | 24.7 |  |  |  |  |
| Center | 17.2 | 19.1 |  |  |  |  |
| South | 39.6 | 31.6 |  |  |  |  |

^1^ Range: from -5.76 to 16.38

^2^ Range: from -5.04 to 27.89

**Supplementary Table 2 – Incidence rate ratio (IRR 95% CI) of cardiovascular diseases (CVD) by quartile of Occupational Physical Activity (OPA), stratified by Leisure Time Physical Activity (LTPA).**

|  | **Mortality** | | **CVD** | |
| --- | --- | --- | --- | --- |
|  | **LTPA no^1^** | **LTPA yes^2^** | **LTPA no^1^** | **LTPA yes^2^** |
| **Men** | **IRR (95% CI)** | **IRR (95% CI)** | **IRR (95% CI)** | **IRR (95% CI)** |
| **Ergo index** (ref: 1° quartile) | 1 | 1 | 1 | 1 |
| 2° quartile | 1.12  (0.61 – 2.05) | 0.98  (0.55 - 1.77) | 1.24  (0.94 – 1.64) | 1.11  (0.87 – 1.43) |
| 3° quartile | 1.39  (0.78 – 2.47) | 1.40  (0.81 – 2.43) | 1.28  (0.97 – 1.68) | 1.17  (0.92 – 1.50) |
| 4° quartile | 1.20  (0.69 – 2.09) | 1.52  (0.91 – 2.54) | 1.07  (0.83 – 1.38) | 1.06  (0.84 – 1.33) |
| **Women** | **IRR (95% CI)** | **IRR (95% CI)** | **IRR (95% CI)** | **IRR (95% CI)** |
| **Ergo index** (ref: 1° quartile) | 1 | 1 | 1 | 1 |
| 2° quartile | 0.43  (0.18 – 0.99) | 0.82  (0.35 - 1.88) | 1.04  (0.73 – 1.47) | 0.91  (0.62 – 1.32) |
| 3° quartile | 0.81  (0.46 – 1.42) | 0.66  (0.31 – 1.39) | 1.34  (1.02 – 1.77) | 1.24  (0.94 – 1.62) |
| 4° quartile | 0.68  (0.37 – 1.23) | 0.89  (0.42 – 1.90) | 1.11  (0.83 – 1.50) | 1.31  (0.97 – 1.76) |

* p<0.05, ** p<0.01.

^1^ No physical activity.

^2^ Light + regular + intense physical activity.

^3^ Adjusted for age, household typology, household economic resources, Physical Component Summary, geographical area of residence, educational level, BMI, pack-years smoking, diabetes, hypertension.

**Supplementary Table 3 – Incidence rate ratio (IRR 95% CI) of mortality and cardiovascular diseases (CVD) by quartile of Occupational Physical Activity (OPA) and gender in the TLS 2001 cohort after exclusion of subjects present also in the TSL 2011 cohort.**

|  | **Mortality** | | **CVD** | |
| --- | --- | --- | --- | --- |
|  | **MEN** | **WOMEN** | **MEN** | **WOMEN** |
| **Men** | **IRR (95% CI)** | **IRR (95% CI)** | **IRR (95% CI)** | **IRR (95% CI)** |
| **Ergo index** (ref: 1° quartile) | 1 | 1 | 1 | 1 |
| 2° quartile | 1.11  (0.99 – 1.25) | 1.01  (0.86 - 1.18) | 1.02  (0.95 – 1.10) | 1.31  (1.20 – 1.42) |
| 3° quartile | 1.04  (0.92 – 1.17) | 0.98  (0.85 – 1.13) | 1.01  (0.94 – 1.09) | 1.16  (1.07 – 1.26) |
| 4° quartile | 1.14  (1.02 – 1.27) | 1.23  (1.05 – 1.44) | 0.98  (0.91 – 1.06) | 1.34  (1.22 – 1.47) |

^3^ Adjusted for age, household typology, Charlson Index, educational level, are deprivation index.


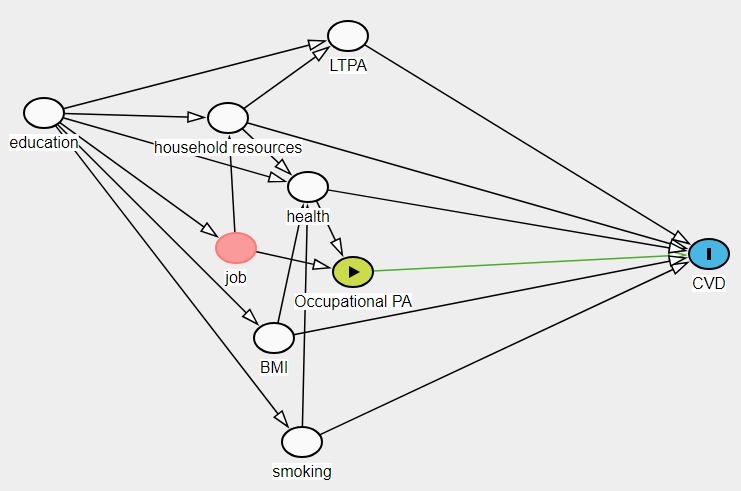


**Supplementary Figure 1. Simplified DAG of the total effect of occupational physical activity on cardiovascular diseases (CVD).** Nodes: Green (with ►) = exposure, Blue (with I) = outcome, Red = ancestor of exposure and outcome (confounder), White = adjusted variable. Arrows: Green = causal path, black = blocked path. Abbreviation: Occupational PA=Occupational physical activity. LTPA=leisure time physical activity. BMI=body mass index.
